# State-of-the-art CT and MR imaging and assessment of atherosclerotic carotid artery disease: the reporting—a consensus document by the European Society of Cardiovascular Radiology (ESCR)

**DOI:** 10.1007/s00330-022-09025-6

**Published:** 2022-10-04

**Authors:** Luca Saba, Christian Loewe, Thomas Weikert, Michelle C. Williams, Nicola Galea, Ricardo P. J. Budde, Rozemarijn Vliegenthart, Birgitta K. Velthuis, Marco Francone, Jens Bremerich, Luigi Natale, Konstantin Nikolaou, Jean-Nicolas Dacher, Charles Peebles, Federico Caobelli, Alban Redheuil, Marc Dewey, Karl-Friedrich Kreitner, Rodrigo Salgado

**Affiliations:** 1 Department of Radiology, University of Cagliari, Cagliari, Italy; 2 Division of Cardiovascular and Interventional Radiology, Department of Biomedical Imaging and Image-Guided Therapy, Medical University of Vienna, Vienna, Austria; 3 Department of Radiology, University Hospital Basel, University of Basel, Basel, Switzerland; 4 BHF Centre for Cardiovascular Science, University of Edinburgh, Chancellor’s Building, 49 Little France Crescent, Edinburgh, EH164SB UK; 5 Edinburgh Imaging Facility QMRI, University of Edinburgh, Edinburgh, UK; 6 Policlinico Umberto I, Department of Radiological, Oncological and Pathological Sciences, Sapienza University of Rome, Rome, Italy; 7 Department of Radiology & Nuclear Medicine, Erasmus MC, Rotterdam, The Netherlands; 8 Department of Radiology, University of Groningen, University Medical Center Groningen, Hanzeplein 1, 9713 GZ Groningen, The Netherlands; 9 Department of Radiology, Utrecht University Medical Center, Heidelberglaan 100, 3584 CX Utrecht, The Netherlands; 10 Department of Biomedical Sciences, Humanitas University, Via Rita Levi Montalcini 4, 20072 Pieve Emanuele, Milan, Italy; 11 IRCCS Humanitas Research Hospital, Via Manzoni 56, 20089 Rozzano, Milan, Italy; 12 Department of Radiological Sciences - Institute of Radiology, Catholic University of Rome, “A. Gemelli” University Hospital, Rome, Italy; 13 Department of Diagnostic and Interventional Radiology, University of Tuebingen, Tübingen, Germany; 14 Department of Radiology, Normandie University, UNIROUEN, INSERM U1096 - Rouen University Hospital, F 76000 Rouen, France; 15 Department of Cardiothoracic Radiology, University Hospital Southampton, Southampton, UK; 16 University Clinic of Nuclear Medicine Inselspital Bern, University of Bern, Bern, Switzerland; 17 Institute of Cardiometabolism and Nutrition (ICAN), Paris, France; 18 Department of Cardiovascular and Thoracic, Imaging and Interventional Radiology, Institute of Cardiology, APHP, Pitié-Salpêtrière University Hospital, Paris, France; 19 Laboratoire d’Imagerie Biomédicale, Sorbonne Universités, UPMC Univ Paris 06, INSERM 1146, CNRS 7371, Paris, France; 20 Department of Radiology, Charité - Universitätsmedizin Berlin, Charitéplatz 1371, 10117 Berlin, Germany; 21 Department of Diagnostic and Interventional Radiology, University Medical Center, Mainz Langenbeckstraße 1, 55131 Mainz, Germany; 22 Department of Radiology, Antwerp University Hospital & Antwerp University, Holy Heart Lier, Berlaar, Belgium

**Keywords:** Carotid artery diseases, Consensus, CT angiography, MR, Atherosclerotic plaque

## Abstract

**Abstract:**

The European Society of Cardiovascular Radiology (ESCR) is the European specialist society of cardiac and vascular imaging. This society’s highest priority is the continuous improvement, development, and standardization of education, training, and best medical practice, based on experience and evidence. The present intra-society consensus is based on the existing scientific evidence and on the individual experience of the members of the ESCR writing group on carotid diseases, the members of the ESCR guidelines committee, and the members of the executive committee of the ESCR. The recommendations published herein reflect the evidence-based society opinion of ESCR. The purpose of this second document is to discuss suggestions for standardized reporting based on the accompanying consensus document part I.

**Key Points:**

*• CT and MR imaging-based evaluation of carotid artery disease provides essential information for risk stratification and prediction of stroke.*

*• The information in the report must cover vessel morphology, description of stenosis, and plaque imaging features.*

*• A structured approach to reporting ensures that all essential information is delivered in a standardized and consistent way to the referring clinician.*

**Supplementary Information:**

The online version contains supplementary material available at 10.1007/s00330-022-09025-6.

## Introduction and purpose of this document

In the last 20 years, new evidence has been added to the understanding of the physiopathology of the carotid-related stroke occurrence, by introducing the concept of the carotid artery vulnerability related to the plaque’s features. At the same time, a significant evolution in the imaging techniques has occurred by routinely allowing not only the degree of stenosis quantification but also the assessment of the plaque composition and the detection of the features of vulnerability.

The European Society of Cardiovascular Radiology (ESCR) is the European specialist society of cardiac and vascular imaging. This society’s highest priority is the continuous improvement, development, and standardization of education, training, and best medical practice, based on experience and evidence. The present intra-society consensus is based on the existing scientific evidence and on the individual experience of the members of the ESCR writing group on carotid diseases, the members of the ESCR guidelines committee, and the members of the executive committee of the ESCR. The recommendations published herein reflect the evidence-based society opinion of ESCR. The purpose of this second document is to discuss suggestions for standardized reporting based on the accompanying consensus document part I.

## Standardized reporting

***ESCR Consensus Statement***

*In reporting carotid artery atherosclerotic disease, assessment of stenosis severity should be complemented with information regarding plaque morphology and plaque composition. Some imaging biomarkers, described in the accompanying part I document, which are not yet validated by official guidelines should be reported and interpreted with caution.*

In this section, we will explain how and what information should be included in the report for the assessment of carotid artery pathology. We classified the information as [mandatory] and [*optional*]. Some information is modality-specific (e.g., only MR). For broad application, this document does not include advanced approaches that are not considered standard or proprietary technologies.

In general terms, a carotid imaging CT/MR report has three main components: (1) description of presence and degree of stenosis, (2) description of the different plaque components, and (3) description of vessel characteristics.

Also, both sides need to be evaluated and commented on, not limiting the analysis to the initially targeted vessel. Finally, the whole trajectory of the internal carotid arteries needs to be evaluated, including the intracranial segments.

Table 1 provides a proposal for structured reporting applicable for both CT and MR examinations. A glossary of commonly used terms is also added as supplemental material.

**Table 1:** CT/MR structured reporting proposal. *NASCET*, North America Symptomatic Carotid Endarterectomy Trial; *PAU*, penetrating atherosclerotic ulcer; *FMD*, fibromuscular dysplasia

**Feature**	**Report component**	**Comments and practical recommendations**
**Stenosis**		
Degree	% luminal stenosis	NASCET-method for stenosis grading recommended
		Consider the narrowest luminal diameter for the calculation of stenosis
		Perform measurements in a plane perpendicular to vessel long-axis (double-oblique image)
		Reference diameter must be unaffected (consider underfilling, near-occlusion)
Location	Bulbar	Atherosclerotic stenoses are usually located at the carotid sinus
	Supra-bulbar	For supra-bulbar extracranial lesions, consider an alternative diagnosis (e.g. FMD)
	Skull base	
	Intracranial	
Tandem stenosis present	Yes/no	Describe location and severity
**Plaque components**		
Plaque surface	Smooth	Presence of irregular contour/ulceration is associated with increased stroke risk
	Irregular	
	Ulceration	
Type of plaque	Low-attenuated/non-calcified	CT: Low-attenuated: <60 HU; non-calcified: 60-130 HU; Calcified: > 130 HU
	Mixed	Describe also the relative contribution of different components (e.g. large non-calcified plaque with small spotty calcification)
	Calcified	
Remodelling	Type	Adequately describe the impact of the plaque morphology and expansion on the vessel lumen
	Eccentricity index	
Calcification	Severity	Absent, mild, moderate, severe
	Morphology	Bulky, (semi)circular, spotty.
Signs of inflammation	Perivascular fat infiltration	Not routine, mention when detectable
	neovascularisation	Not routine
Intraplaque hemorrhage	yes/no	MR-preferred
**Vessel characteristics**		
Aortic arch morphology	Gross arch anatomy	Consider arch atherosclerosis as a source for thromboembolic events, especially in the presence of PAU
	Branching pattern	Branching pattern and aortic arch morphology may influence therapeutic strategy (surgery vs stent)
	Presence and degree of atherosclerosis	
Morphology carotid arteries	Unremarkable	
	Elongation	
	Kinking	
	Coiling	
Vertebral arteries	Normal/abnormal	Describe abnormalities when present
	Dominance	
Basilar artery	Normal/abnormal	
Circle of Willis	Anatomic variants	
	Communication between anterior and posterior circulation	
**Ancillary findings**		
	Describe any abnormal findings in other visualized structures/anatomical regions. However, consider that the contrast phase of CT/MR acquisition is usually the arterial phase only.	

### Degree of stenosis [mandatory, CT/MR]

As previously stated, we recommend the NASCET method to quantify ICA stenosis. The narrowest luminal diameter at the level of the stenosis must be used for the calculation of stenosis severity. Measurements should be performed in a plane perpendicular to the vessel’s long axis. This implies the construction of a CMPR. Standard, axial, sagittal, or coronal planes are not applicable. Also, the used reference diameter distal from the stenosis should be a patent vessel segment.

**Fig. 1:**
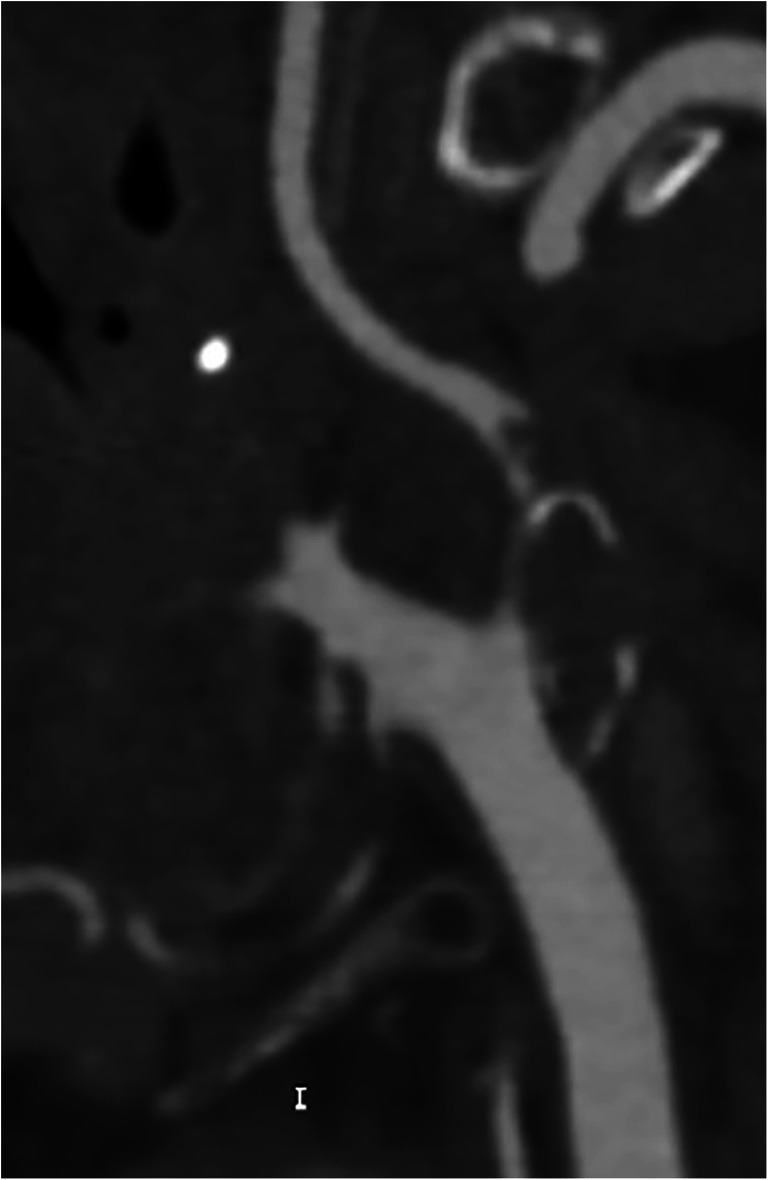
Near-occlusion in the internal carotid artery bulb due to an extensive embolic and now thrombosed fragment. The correct term for this sub-occluded lumen with the collapse of the distal portion of the ICA is “near-occlusion” and not “99% stenosis.” In this case, the distal portion of the ICA as the denominator for the quantification of the degree of stenosis should not be used

The fact that the NASCET method uses the normal distal ICA for the calculation of the stenosis percentage has some practical implications that one should be aware of. First, caution should be taken in cases of near-occlusion with post-stenotic underfilling [1, 2]. Therefore, the NASCET collaborators recommend assessing near-occlusion first, as in such cases a collapsed/underfilled distal ICA used for percentage calculation will result in an underestimation of stenosis severity (Fig. 1). As such, it is an error to report a near-occlusion as 99% stenosis as in this specific situation the distal portion of the ICA may not be used as a denominator for the quantification of the degree of stenosis [1, 2].

**Fig. 2:**
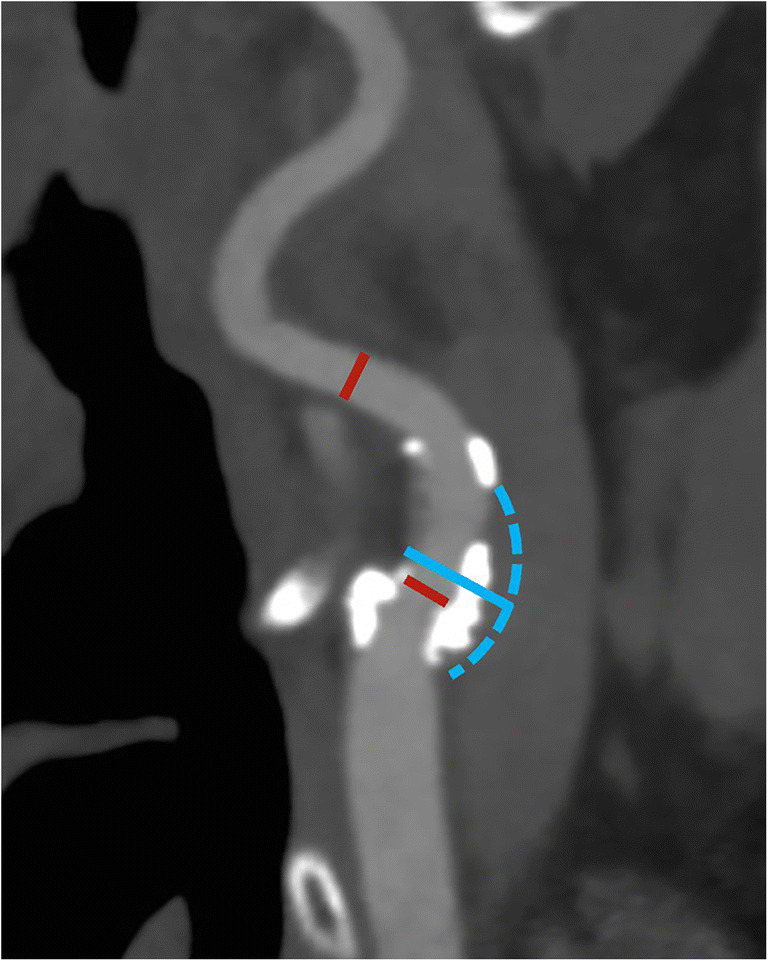
Differences in degree of stenosis between NASCET and ESCT. In this patient, a large mostly calcified plaque can be found in the bulb of the internal carotid artery. However, the luminal diameter remains unchanged compared with a reference diameter beyond the carotid bulb (red lines), resulting in a 0% NASCET-stenosis. However, this does not equal the absence of carotid disease. Conversely, the ESCT-measured stenosis is 52%, as the outer diameter of the carotid bulb (blue solid and dashed line) as reference diameter meter reflects the current situation. In situations like this, a detailed reporting of plaque location and characteristics is essential to accurately describe the findings beyond the degree of luminal narrowing

**Fig. 3:**
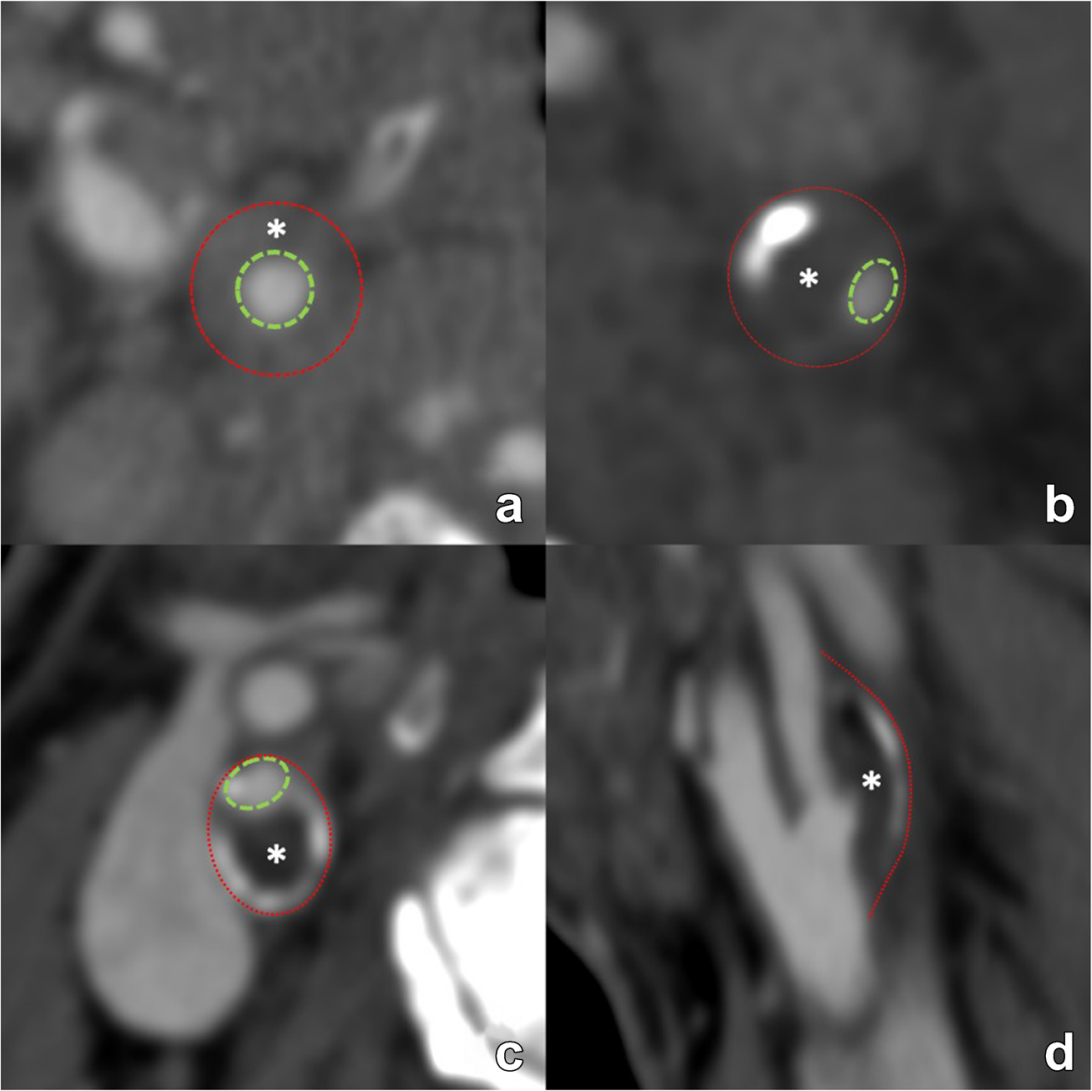
Various examples of arterial remodeling are illustrated. First, two examples of inward/negative remodeling are shown (**a**, **b**). The outer diameter of the artery (red dotted line) remains in both examples unchanged. In both cases, the lumen is narrowed (green dotted line) due to a plaque (asterisk), indicating negative/inward remodeling. However, due to the concentric morphology of the plaque in panel a, the narrowed lumen remains in a central position, representing concentric remodeling. Conversely, in panel b the luminal narrowing is eccentric. Panels **c** and **d** illustrate outward/positive eccentric remodeling, where the outer perimeter of the artery, outlined by the red dotted line, is increased due to plaque formation with eccentric luminal narrowing. The positive remodeling example illustrates the notion that significant plaque can be present but still produce only mild stenosis (only 25% NASCET-calculated stenosis in this case) with a mostly outward protruding plaque. NASCET: North American Symptomatic Carotid Endarterectomy Trial

Second, merely reporting the degree of luminal stenosis can result in the underestimation of carotid disease as the luminal diameter can be maintained through the positive remodeling of the vessel wall with outward growth and without luminal compromise rather than inward growth with stenosis. This is further illustrated in the NASCET method by the anatomy of the carotid artery at the level of the bulb, as the normal outward bulge of the lumen at the carotid bulb may be lost due to the presence of disease but still not cause any measured stenosis given the location of the reference diameter (Fig. 2). Consequently, the presence of positive/negative remodeling should always be reported as well (Fig. 3). While both CT and MR can accurately visualize remodeling, it in practice easier to assess on CT given among others the inherent 3D isotropic nature of the image acquisition.

Finally, special care should be taken to identify possible tandem stenosis, defined as the simultaneous presence of severe carotid bifurcation stenosis and second stenosis of ≥50% in any downstream distal cerebral artery. This includes the intracranial arteries and the circle of Willis.

### Plaque components and morphology

An overview of the different plaque components, their clinical significance, and preferred imaging modality is given in Table 2.

**Table 2:** A overview of different plaque components, their clinical significance, and preferred imaging modality

Plaque feature	Pathophysiological impact	Feature of plaque vulnerability	Preferred imaging modality	Reported in clinical practice
Type of plaque	See below for comments on specific components		CT	Yes
Fibrous cap	Thinning of the fibrous cap increases the risk of plaque rupture	++	MR	No
Calcifications	While overall considered a sign of a plaque stability, conversely the type and chemical composition of calcium in atheromatous plaques can potentially increase plaque vulnerability	(debated)	CT	Yes
Intraplaque hemorrhage	Most important imaging biomarker for plaque instability. It is independent of stenosis severity, associated with acute events, and also with an increased risk for ipsilateral future ischemic events in both symptomatic and asymptomatic subjects.	+++	MR	Yes
Plaque ulceration	Presence of ulcerations is associated with cerebrovascular events	++	CT	Yes
Lipid-rich necrotic core	An increased amount of intraplaque lipid is associated with elevated cerebrovascular risk.	++	MR	Yes
Maximum Wall thickness	Maximum plaque thickness (measured in mm) is a predictor of cerebral ischemic events	+	CT/MR	Research setting
Plaque volume	Larger volume is associated with increased vulnerability and occurrence of future cerebrovascular events	+	CT>MR	Research setting
Plaque neovascularisation	Denser vasa vasorum network is associated with symptomatic disease	+	CT/MR	Research setting
Plaque- and perivascular fat inflammation	Inflammation is associated with cerebrovascular events	+++	^18^F-FDG PET-CT / MR	Research setting
Plaque remodelling	Remodelling refers to cross-sectional vessel area changes in reaction to atherosclerotic changes. Inadequate outward (positive) eccentric remodelling is associated with symptomatic disease and increased ipsilateral cerebrovascular events.	++	CT/MR	Yes

#### Type of plaque [mandatory, CT]

**Fig. 4:**
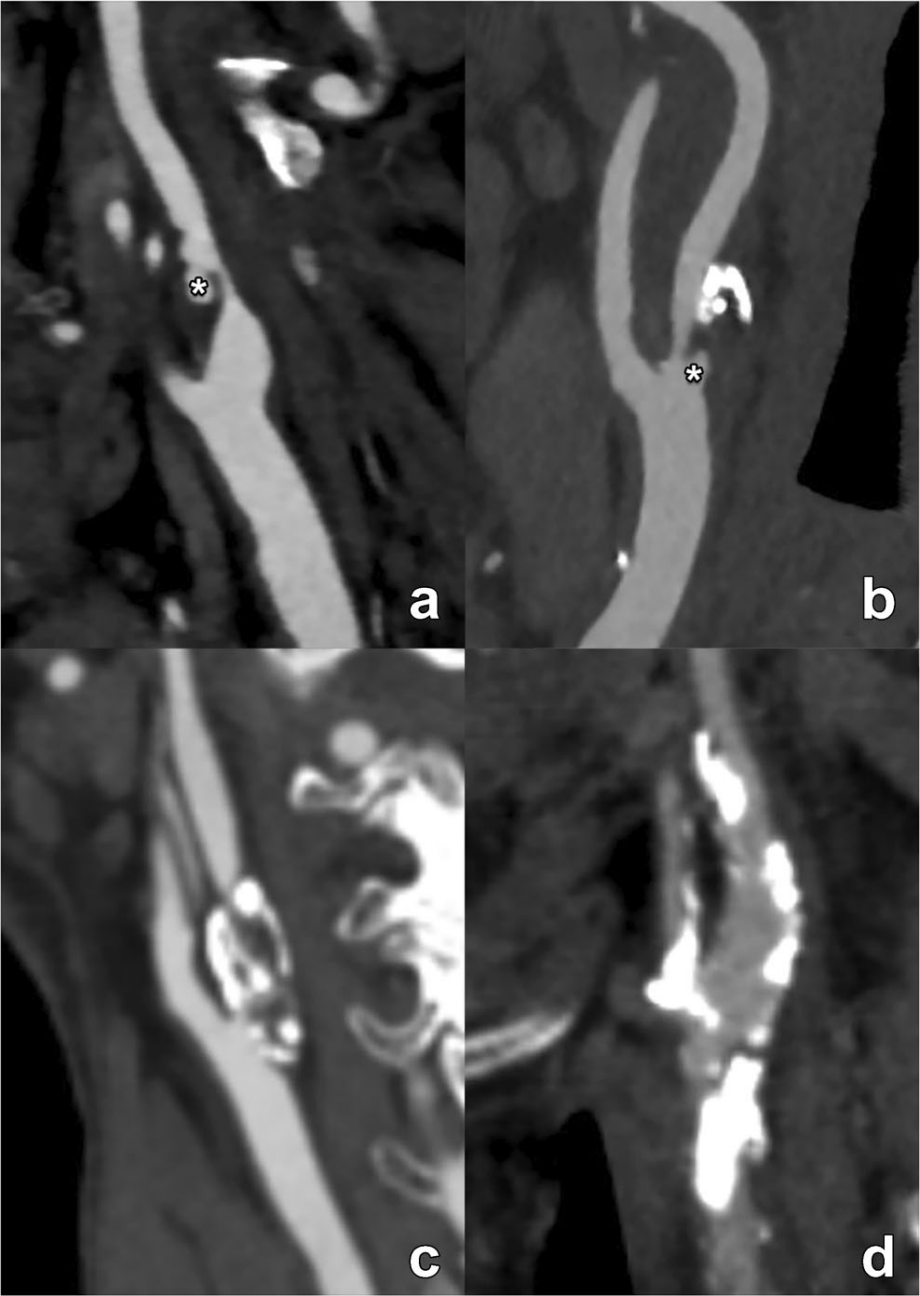
Varying presentation of carotid plaques with different ratios of calcified and non-calcified components (**a**–**d**). The different panes illustrate several plaque configurations ranging from a completely non-calcified (**a**) to an extensively and exclusively calcified plaque (**d**) with different calcified/non-calcified ratios in between (**b**, **c**). Note that, independently of the plaque composition and the degree of luminal stenosis, panel a and b reveal additional ulcerations (asterisk), a plaque feature with prognostic implications which must certainly be mentioned in the report. These examples further illustrate the notion that the description of carotid plaques must extend beyond mere calculation of luminal stenosis, mentioning all relevant plaque features to provide the most complete disease assessment

The fundamental CT analysis of the type of the plaque is based on the attenuation value. According to the HU attenuation values it should be described as low-attenuated (< 60 HU), mixed (between 60 and 130 HU) or calcified (> 130 HU) plaques (Fig. 4). These values however vary with chosen tube voltage and the presence of iodinated contrast. A setting of 120 kV is recommended for plaque density measurement. Also, while CT offers the possibility of quantitative evaluation of some plaque features, the accuracy of these measurements is very closely linked to the obtained image quality. Any degradation of the latter, e.g. due to the presence of previous dental work or any other source of artifacts, can compromise the CT measurements and lead to the wrong conclusion.

#### Plaque composition: calcification [mandatory, CT]

**Fig. 5:**
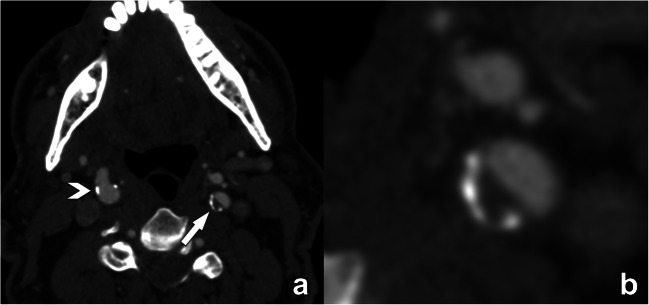
Illustration of the “rim”-sign on contrast-enhanced CT. The axial CT-image at the level of the internal carotid artery reveals only minimal small calcifications in the right ICA (arrowhead in **a**), and a plaque with calcified and non-calcified components in the left ICA (arrow in **b**). Close examination of this plaque reveals a small semicircular rim of adventitial calcifications at the periphery of the plaque (<2 mm thickness) with a larger (>2 mm) non-calcified plaque component bordering the lumen. This plaque phenotype is known as a positive “rim”-sign, a feature associated with the presence of intraplaque hemorrhage and as such with higher stroke risk. Reporting of this type of plaque is significant, as the luminal narrowing is only moderate and by itself according to NASCET criteria considered non-significant

The presence and morphology of calcification should be described. The severity should be visually assessed and broadly described as absent, mild, moderate, or severe (grade 0, 1, 2, or 3). The presence of a positive “rim sign” (Fig. 5), defined as the presence of an adventitial calcification (<2-mm thick) with internal non-calcified plaque (≥2-mm thickness), should be reported because of its potential association with IPH.

Dual-energy CT with post-processing using 80–100 keV provides the most accurate estimates of calcification size, as compared to histology[3] [*optional*].

#### Plaque composition: intraplaque hemorrhage [mandatory, MR]

**Fig. 6:**
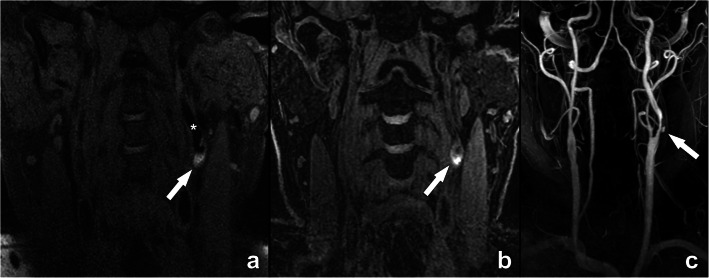
Intraplaque hemorrhage (IPH) demonstrated on MR in a 74-year-old man with a left-sided stroke. This case of intraplaque hemorrhage clearly demonstrates the T1-shortening effect of methemoglobin generated from erythrocyte degradation, illustrated as an intralesional hyperintense signal (arrows in a, b) in the left internal carotid artery (asterisk in **a**), as shown in CUBE (**a**) and MPRAGE-sequences (**b**). For additional evaluation of luminal repercussion, a contrast-enhanced MR angiography sequence was executed, revealing a severe luminal narrowing with a focal ulceration (arrow in **c**). The presence of plaque ulceration and intraplaque hemorrhage are two important features in this case that provide additional clinically and prognostically relevant information on top of the mentioned luminal narrowing. CUBE: General Electric proprietary name for a 3D fast spin echo sequence; MPRAGE: magnetization-prepared 180° radio-frequency pulses and rapid gradient echo

With MR, the identification of intraplaque hemorrhage (IPH) is possible using different approaches. The simplest approach is the detection of focal regions of T1 hyperintensity within the carotid plaque that is 1.5 times greater than the adjacent sternocleidomastoid muscle, attributable to the strong T1-shortening effect of methemoglobin generated from erythrocyte degradation (Fig. 6) [4]. It is also possible to use the time-of-flight (TOF) for IPH detection [5] due to its inherent T1 contrast. Limitations include susceptibility to flow artifacts, particularly in tortuous segments, low sensitivity to IPH, and inability to differentiate juxtaluminal IPH from ulceration [6].

CTA is not yet considered a robust method to detect the presence of IPH but several studies suggest that some specific features are significantly associated with the presence of IPH in CTA: presence of the napkin sign [7], positive rim sign [8], and presence of internal low attenuation (< 25 HU or 30 HU according to different groups) [9, 10].

#### Presence of remodeling [mandatory, CT/MR]

Remodeling should be classified as outward (positive), neutral, or inward (negative) [*mandatory*] (Fig. 3). It is also possible to add the plaque remodeling ratio (RR) that is determined, according to Hardie [11], by (1) measurement of the outside vessel circumference of the extracranial internal carotid artery at the point of maximal luminal stenosis, and (2) divided by the outside vessel circumference at a region unaffected by atherosclerotic disease [*optional*]

The eccentric or concentric morphology of the plaque must be at least visually evaluated [*mandatory*]. Alternatively, it can be expressed quantitatively with the eccentricity index (EI) of the plaque calculated as the ratio between the maximum thickness and minimum thickness at the point of maximum stenosis [12] [*optional*]

#### Plaque ancillary findings: thrombus (floating) [mandatory]

**Fig. 7:**
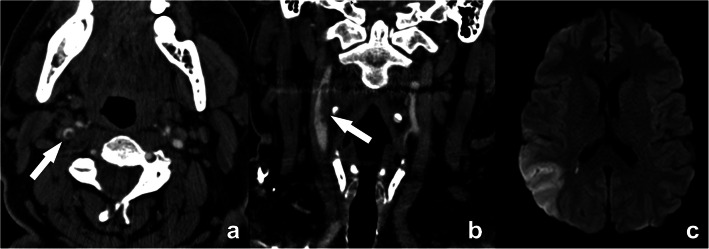
Floating thrombus in the supra-bulbar right internal carotid artery. A large eccentric non-calcified thrombus is clearly seen, on cross-sectional CTA images appearing as a central non-enhancing thrombus surrounded by a (semi-)circular enhancing lumen, the so-called “donut”-sign (arrow in **a**). A coronal CTA image further reveals the irregular outer border of this floating thrombus (arrow in **b**), indicative of a higher risk for distal emboli. This is further illustrated by an axial diffusion-weighted MR-image of the same patient revealing a recent ischemic infarct in the distribution region of the right media cerebral artery

Intraluminal thrombus, an additional marker that is highly associated with stroke [13], can be detected on CTA as a donut sign (Fig. 7) [14]. Another definition is the presence of a filling defect outlined by lumen contrast that is visualized on multiplanar reformats. Both CT and MR are able to detect thrombus.

#### Plaque ancillary findings: carotid web [mandatory]

**Fig. 8:**
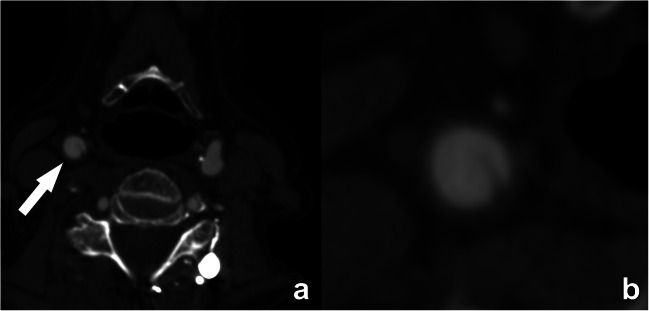
Carotid web in the right internal carotid artery. A semi-circular non-calcified thin lesion is seen protruding in the lumen from the posterior wall of the artery (arrow in **a**). No calcification or thrombus can be seen, as further illustrated in the detailed image (**b**). Some authors consider a carotid web to be a variant of fibromuscular dysplasia. A carotid web may induce flow stasis with thrombus formation, hence increasing the risk of stroke

A carotid web appears as a shelf-like projection into the lumen of the proximal cervical internal carotid artery without evidence of calcification (Fig. 8) [15]. This is a proposed stroke mechanism that may underlie cryptogenic stroke, particularly in younger patients without vascular risk factors [16].

#### Plaque composition: lipid-rich necrotic core [optional, MR]

As previously stated, CT cannot assess the presence of lipid-rich necrotic core (LRNC) but can identify the presence of low-density areas that could represent LRNC. Basic assessment involves measuring attenuation density in a ROI in the plaque. More advanced assessment involves the calculation of the volume of plaque subcomponents.

MR T2-weighted imaging can be used to detect the presence of LRNC [17]. Direct assessment of LRNC can also be done in patients undergoing contrast administration using a post-contrast T1w scan. CE-MRA followed by post-CE vessel wall imaging in patients without contraindication will improve the detection and quantification of the LRNC.

#### Status of the fibrous cap [optional, MR]

When assessing the carotid plaque with high-resolution MRI, information on the fibrous cap status should be given by categorizing it as thick (normal), thin, or fissured [18].

#### Plaque inflammation [optional, CT/MR]

It is possible to include information with CT, regarding the perivascular fat density (PFD) measured as the attenuation value of the pericarotid fat. It should be measured as a circular/elliptic ROI obtained close to the point of maximum stenosis [*optional*].

Ultrasmall superparamagnetic particles of iron oxide enhanced MRI has been shown to identify macrophage infiltration [19].

PET/CT or PET/MR can be used to study plaque inflammation or biological activity, along with plaque characterization in a single examination [20]. However, only 18F-FDG PET is to date routinely used in clinical practice, and when assessing small structures, the sensitivity and specificity can be low due to spatial resolution and often suboptimal lesion-to-background ratio. New tracers that are being developed are warranted to enhance the role of PET/CT and PET/MR in clinical practice.

#### Plaque neovascularization [optional]

It is possible to include information about plaque neovascularization on CT, with the contrast plaque enhancement (CPE) value by measuring the attenuation value of the plaque with a region of interest (ROI) on pre- and post-contrast scans. It is important to avoid areas of calcium and beam hardening, because of the high attenuation value, and to compare exactly the same ROI on the pre- and post-contrast imaging.

Using MR, the detection and quantification of neovascularization are possible using the DCE-MR (K^trans^ - volume transfer coefficient method). Alternatively, gadofosveset-enhanced MRI can be used to visualize plaque microvasculature without the need to use pharmacokinetic modeling [21].

#### Plaque burden—distribution and subcomponents [optional]

Knowledge of the location and distribution of plaques can be helpful in the pre-procedural workup of a patient with carotid artery disease. In particular, plaque length measurement (longitudinal extension) and the relationship of a targeted plaque with the carotid bifurcation may provide better insights regarding the optimal surgical approach or the possibility to deploy a stent. This information can be easily obtained using CPR reconstructions along the trajectory of interest with centerline measurements, usually performed on CT but also possible with MR. Moreover, CT can calculate the volume of carotid artery plaque and determine the volume of plaque sub-components, according to the attenuation density threshold.

With MR, efficient 3D large coverage black-blood MR may be better suited for this purpose. With commercially available software it is possible to obtain a fast visualization of the spatial localization of the tissue components.

While this information is not currently a parameter required in standard CT/MR reports due to the lack of standardization and required post-processing, it may be implemented on a case-by-case basis. Also, it is reasonable to think that with the current ongoing evidence related to the impact of the plaque burden and the development of advanced software packages, this parameter could also be incorporated into the future radiological report on the carotid arteries[22].

### Vessel morphology [all sections mandatory]

Most atherosclerotic plaques occur at the carotid bifurcation, involving the distal CCA and the bifurcation and extending to the proximal ICA. Nevertheless, a detailed analysis should be performed of the complete trajectory of the carotid arteries from their origin until their intracranial segments.

#### Definition of the aortic arch and supra-aortic vessel branching anatomy

The description of the aortic arch [including the presence of calcification, aneurysm, thrombi, and/or penetrating atherosclerotic ulcers (PAU)] as well as *branching* anatomy of supra-aortic vessels and the presence of subclavian artery pathology is fundamental given the potential implications for carotid artery treatment.

#### Common carotid artery status

The presence and quantification of the severity of plaque (> 50%) in the common carotid artery (CCA) should be reported by describing the type (calcified, low-attenuated, mixed).

#### ICA vessel tortuosity

Vessel tortuosity can be classified according to the modified criteria of Weibel-Fields and Metz [23–25], which describe the course as tortuous (elongated), kinked (mild, moderate, severe), or coiled when applicable (see also Table 5, part I). This abnormal morphology can be found in all segments of the common carotid artery (CCA) and ICA and should be commented on in both CT and MR reports.

#### Carotid arteries—distal extracranial segment and intracranial segments

Description of any distal plaques should be reported in particular for the potential impact on surgical procedures. The presence of a severe distal carotid artery plaque should be defined as a “tandem” plaque [26]. While the presence of a tandem lesion infrequently alters the surgeon's decision to perform an endarterectomy, detecting tandem stenoses may have important implications for long-term medical management in symptomatic patients [26, 27].

#### Vertebral arteries/basilar and other vessels

Description of anatomy from the subclavian origin through to the basilar artery as well as the presence of at least moderate (> 50%) stenosis. Also, the superior cerebellar artery (SCA), the anterior inferior cerebellar artery (AICA), and the posterior inferior cerebellar artery (PICA) should be commented on in the report when the absence of these vessels is noted.

#### Circle of Willis

Analysis of the circle of Willis should be included. The key information is (1) anatomy with the presence of variants (dominant, absent, or hypoplastic (<1 mm) segments); (2) exclusion of aneurysms; and (3) identification of atherosclerotic (or other types e.g. inflammatory) lesions in the main arteries.

#### Post-intervention imaging

**Fig. 9:**
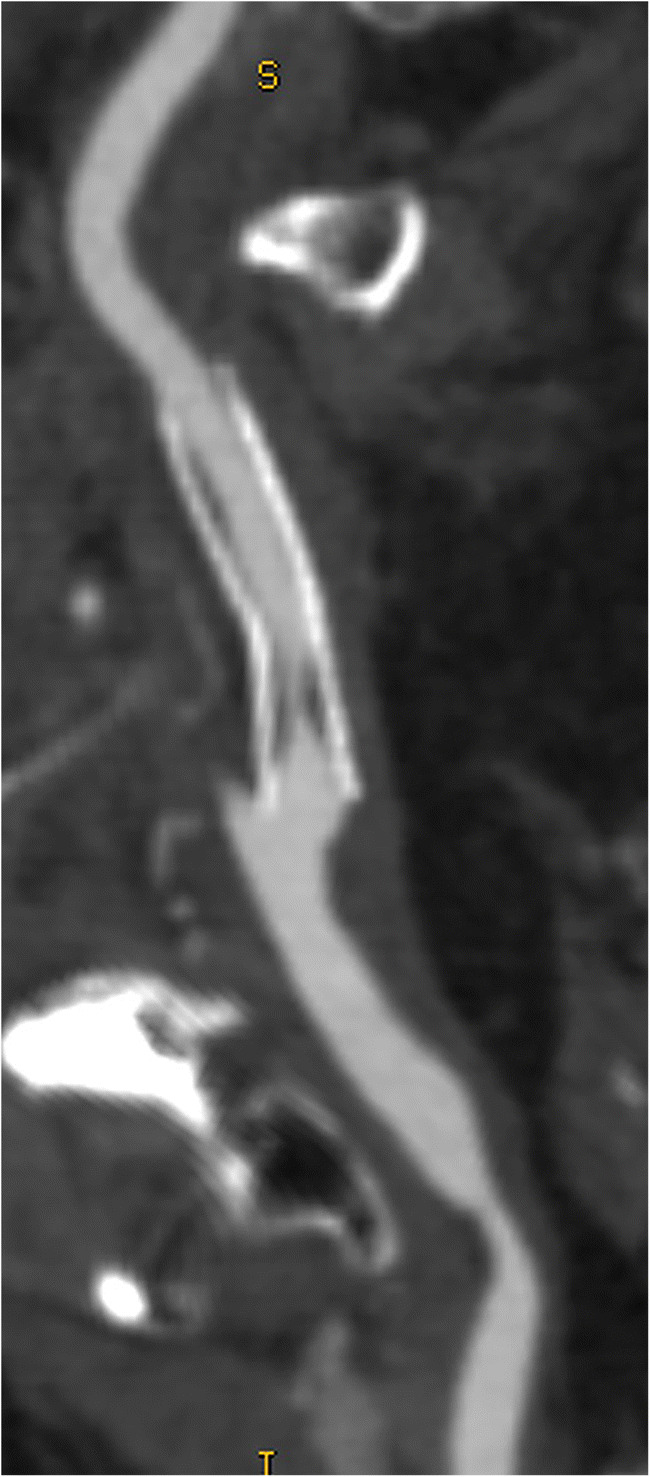
Curved MPR image of neo-intimal hyperplasia in a carotid stent, producing significant proximal luminal narrowing. CT offers exquisite anatomical detail, highlighting complications like the mentioned intima hyperplasia and guiding as such further treatment and potential re-intervention

While the vast majority of CT/MR examinations will be performed for diagnostic purposes, some patients will be referred for imaging after an intervention. Two groups can be distinguished: surgical intervention (carotid endarterectomy or surgical graft placement) versus deployment of a stent. In both instances, the main goal of imaging is the evaluation of the patency of the treated carotid artery segment, and the detection of possible complications. Possible complications include post-operative dissection, neo-intimal stent hyperplasia (Fig. 9), restenosis, and occlusion. While some of these complications may be detected with ultrasound, CT and MR offer greater anatomical detail and deliver all necessary information regarding a possible re-intervention.

### Carotid differential diagnosis

During the assessment of supra-aortic vessels, a differential diagnosis hypothesis can be formulated when typical features are detected. The definition of these multiple entities outside atherosclerotic disease is out of the scope of this paper and only some key elements are further described here.

#### Carotid dissection

**Fig. 10:**
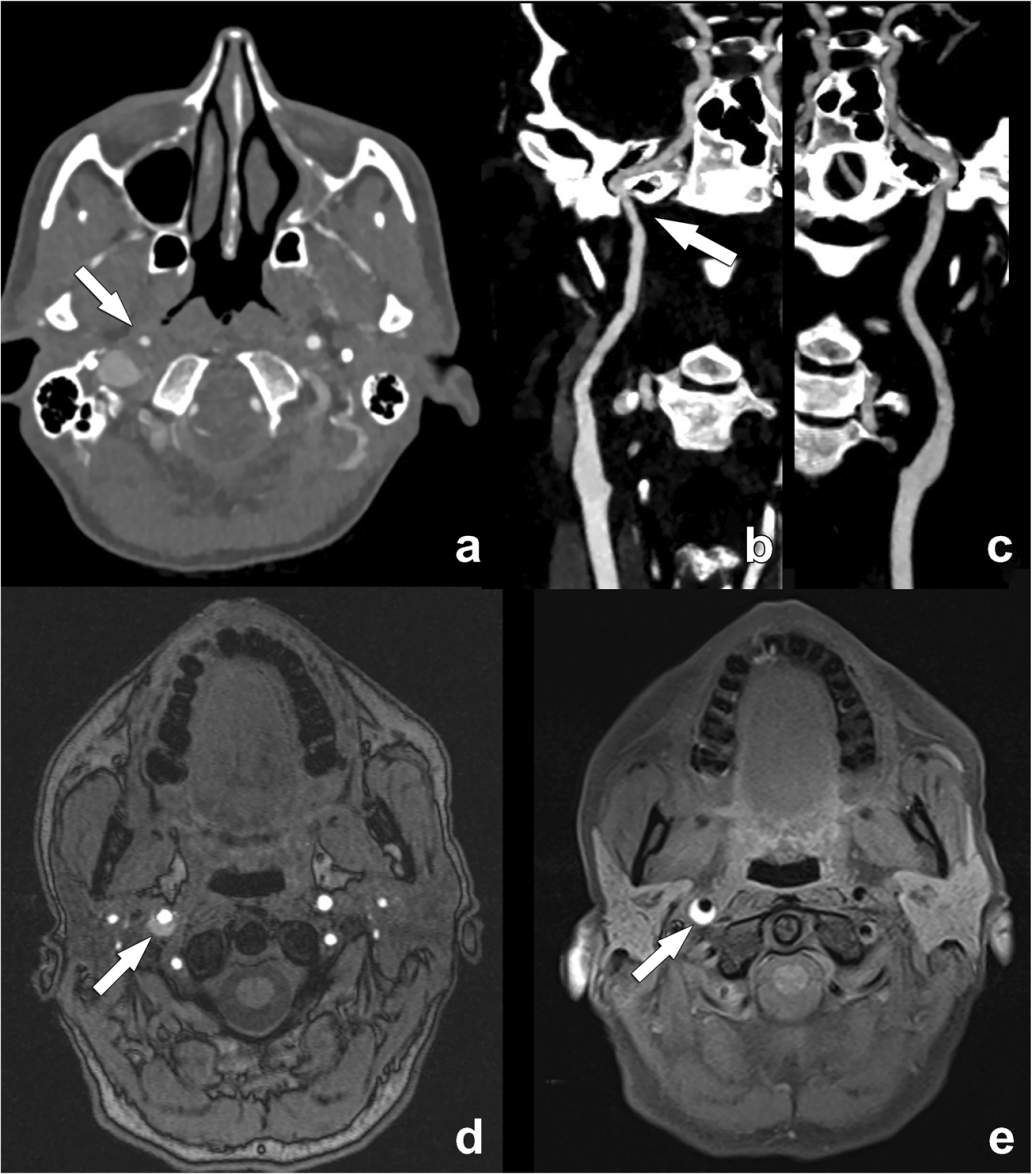
Acute dissection in the distal right internal carotid artery in a 45-year-old woman. The contrast-enhanced CT angiography examination initially revealed a narrowing of the distal extracranial segment of the right ICA (arrow in **a**). Note the absence of an atherosclerotic plaque. While a surrounding soft tissue density could be seen on close examination of the affected artery segment on a professional display, this is often difficult to appreciate. The narrowing is further illustrated on the curved MPR image (arrow in **b**), which can be compared with the normal left ICA (**c**). Due to its ability to visualize blood degradation products, MR is for carotid dissection clearly superior to CT to detect wall hemorrhage. While this can be seen on a TOF sequence (arrow in **d**), a T1-weighted fat-suppressed sequence remains the method of choice to detect the thrombosed false lumen in acute carotid dissection (arrow in **e**)

Carotid dissection is responsible for 20 % of ischemic strokes in young adults under 45 years and for about 2% of ischemic strokes overall [28]. In MR, the detection of this condition is usually straightforward as the blood within the layers (intima and media) has a hyperintense signal visible with T1W-fat-sat sequences (Fig. 10) [29]. In CTA, it is challenging to distinguish the presence of blood degradation products based on the mere attenuation density. Other features that should be identified are as follows: (1) localization, as usually carotid dissection, does not normally involve the bifurcation that is the usual location of atherosclerosis; (2) morphology of the occlusion because the occlusion (or sub-occlusion) usually shows a “flame” sign that is not typical in the atherosclerotic process. In the case of previous dissection, a pseudo-aneurysm can occur, caused by a weakened wall due to the absence of the intima. However, some pitfalls can be encountered such as flow-related enhancement in arteries and veins that can simulate intramural hematoma and special care regarding the technical acquisition is fundamental [30].

#### Fibromuscular dysplasia

**Fig. 11:**
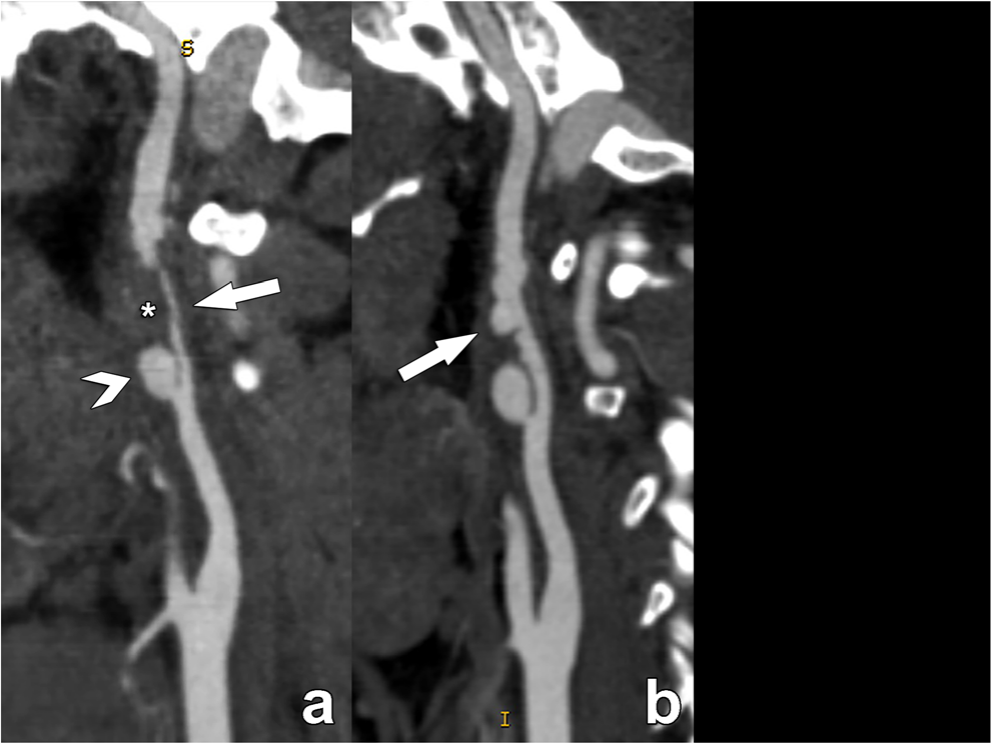
Acute dissection in the supra-bulbar left internal carotid artery (ICA) in a 28-year-old man due to fibromuscular dysplasia, presenting with an acute stroke. The CT angiography examination performed on the day of the acute event reveals a sub-occlusive narrowing of the supra-bulbar left ICA (arrow in **a**), with a large eccentrically located thrombosed part (asterisk in **a**), containing a large proximal pseudo-aneurysm (arrowhead in **a**). Note the complete absence of atherosclerosis in the carotid bulb. On the follow-up examination performed three weeks after the acute event, the thrombus has largely resolved, revealing the typical strings-of-beads sign of fibromuscular dysplasia which was the underlying condition leading to the acute dissection. Fibromuscular dysplasia should always be considered when investigating stroke in young patients

Fibromuscular dysplasia (FMD) is an idiopathic, non-inflammatory, and non-atherosclerotic disease with a prevalence between 0.3 and 3% in the cervico-encephalic arteries. The string-of-beads aspect is highly suggestive of FMD (Fig. 11) [31]. Another frequent finding suggestive of an FMD diagnosis is the presence of a “web-like” defect at the origin of the internal carotid artery [32].

#### Vasculitis

Carotid vasculitis can be defined as the inflammation of carotid artery walls with or without necrosis, leading to stenosis or occlusion of the lumen [33]. Vasculitis may be associated with systemic connective tissue disorders or may be secondary to infection, malignancy, drugs, or radiation therapy. For a correct diagnosis, relevant laboratory tests are also required. The 2012 Chapel Hill Consensus Conference defined different types of vasculitis in terms of (a) the size of the involved arteries and (b) associated pathologic lesions. The most frequent vasculitis involving carotid arteries are Takayasu arteritis and Giant cell arteritis [34] [35]. However, other conditions (e.g., infection, syphilis, tuberculosis, drugs) can also cause vasculitis.

With CTA/MRA imaging, signs of carotid vasculitis are vessel wall thickening (mostly concentric representing a key parameter in the differential diagnosis) and contrast enhancement. Usually, there is no preference for the involvement of the carotid bifurcation (different from atherosclerotic disease). In the case of active vasculitis, contrast enhancement of the thickened vessel wall may be seen on both CT and MR. Some authors suggest using T2-weighted TSE imaging for edema detection in mural inflammation but this is less sensitive and more vulnerable to artifacts compared to T1-weighted, fat-suppressed, contrast-enhanced, black blood MR imaging [36].

#### Presence and treatment of malignancy

Rarely, a solid tumor may be found along the course of the carotid arteries. A carotid body tumor is in typical cases located at the carotid bifurcation, splaying the internal and external carotid arteries but without invading the wall. This typical location together with the hypervascular nature is usually sufficient for a confident diagnosis.

Radiation treatment can also induce non-atherosclerotic luminal narrowing as radiation-induced fibrosis [37]. In a typical case, this presents as a luminal narrowing without plaque formation or remodeling.

#### Ancillary information

The analysis should be completed with the assessment of the other visualized structures [*mandatory*]. All pathological findings should be reported at the end of the report as ancillary findings.

## Discussion and conclusion

The use of standardized and structured reporting for radiology examinations has gained significant momentum in recent years. However, it may come as a surprise that this concept is not new, as Preston Hickey proclaimed almost 100 years ago that “reports filed in hospitals should be scientifically accurate and should follow an accepted nomenclature, so at to have value in a statistical sense” [38].

This formalized approach to delivering the information contained in imaging datasets following a pre-defined template has many advantages. As a standard model sharing the same language, it is a means to avoid errors of interpretation, as the reports will be mostly void of personal preferences in the way they deliver information [39]. Also, it allows the reporting radiologist to follow a step-by-step process, assuring that all relevant information is covered and as such reducing important omissions. This is especially important when radiologists are less experienced in the pathology at hand, especially when performing the examination on call after normal office hours [40].

It is well demonstrated that the simple missing information related to the modality of stenosis degree measurements [41] can determine significant mistakes in the strategies for the section of the therapeutical approach [42].

A standardized and structured reporting could represent an advantage for the use of a correct and reproducible modality of information transfer among the different physicians by using a shared and unified model of presentation and interpretation of the information by reducing the overall frequency of errors [43].

For these reasons, the ESCR has promoted structured reporting in its publications [44] and has a joint venture with specialized companies to promote this among the cardiovascular imaging community [45]. This part II document on carotid imaging presents a further effort to standardize the reporting of carotid pathology in CT and MR examinations and has left room for the inclusion of upcoming technologies that could further change the landscape of plaque imaging.

## Supplementary information

ESM 1 (DOCX 39 kb)

## Abbreviations

^18^F: 18-Fluorodeoxyglucose
AICA: Anterior inferior cerebellar artery
CCA: Common carotid artery
CMPR: Curved multi-planar reformations
CT: Computed tomography
CTA: Computed tomography angiography
EI: Eccentricity index
ICA: Internal carotid artery
IPH: Intraplaque hemorrhage
MR: Magnetic resonance
MRA: Magnetic resonance angiography
NASCET: North American Symptomatic Carotid Endarterectomy Trial.
PD: Proton density
PET: Positron emission tomography
PFD: Perivascular fat density
PICA: Posterior inferior cerebellar artery
SCA: Superior cerebellar artery
SNR: Signal-to-noise ratio
T1W: T1-weighted
TOF: Time of flight
US: Ultrasound
USPIO: Ultrasmall superparamagnetic iron oxide
VR: Volume rendering

## Declarations

### Guarantor

The scientific guarantor of this publication is Luca Saba, Department of Radiology, University of Cagliari, Cagliari, Italy.

### Statistics and biometry

No complex statistical methods were necessary for this paper.

### Methodology

- scientific society consensus statement
