# Supplementary material for: State-of-the-art CT and MR imaging and assessment of atherosclerotic carotid artery disease: the reporting—a consensus document by the European Society of Cardiovascular Radiology (ESCR)
Source: Eur Radiol. 2022 Oct 4;33(2):1088–101. doi: 10.1007/s00330-022-09025-6 (PMC9889425; doi:10.1007/s00330-022-09025-6)
Supplement: Supplementary file 1 — (DOCX 39 kb) [file 330_2022_9025_MOESM1_ESM.docx]

**ESCR - Glossary of terms**

*Members of the ESCR guidelines committee compiled a glossary of terms associated with carotid imaging. Brief descriptions of some diseases and technical concepts are included.*

**Athero-embolism**: consist of cholesterol crystals from the lipid core of a plaque. As such, these are small particles less than 200 µm. It occludes small arterioles and can here trigger an inflammatory process (e.g. blue toe syndrome). Typically, it is a subacute event, bilateral, and occurring in distal vascular territories.

**BB-MRI** (black-blood MR imaging): BBMRI is an MRI sequence that aims at signal suppression from blood flow and therefore allows for a clear delineation of the vessel walls. This is useful for assessing artery walls and plaque features such as IPH.

**Bovine aortic arch:** a term often used to describe an anatomic variant of the aortic arch, in which left common carotid artery origin is moved to the right and merges with the origin of the innominate artery. While the application of this term is widespread, it is however a misnomer, and the description of the anatomy should be used instead as follows: a common origin for the innominate and the left common carotid artery.

**Carotid pseudo- or false aneurysm:** As with pseudoaneurysms elsewhere, they lack all three layers of the arterial wall. Pseudoaneurysm development can occur within hours to several years after initial arterial injury. Most frequent causes are trauma (including endarterectomy) and arterial dissection (e.g. in fibromuscular dysplasia), with infections in a minority of cases. In contrast to true aneurysms, they can grow rapidly with rupture risk unrelated to size. Once detected, urgent intervention is usually required to prevent possible complications such as rupture, embolization, thrombosis, or airway and cranial nerve compression

**Carotid true aneurysm**: True aneurysms are defined as permanent local dilations of an artery involving all three layers (intima, media, adventitia) with at least 50% diameter increase compared to the normal, expected diameter [1]. The thresholds to define the presence of an aneurysm are variable according to the body habitus but in a study with a large population the minimum value considered to define an aneurysm was 0.8 cm [2]. Extracranial carotid aneurysms are rare (1% of all aneurysms). The predominant etiology is atherosclerosis [3]. Uncomplicated cases undergo long-term follow-up.

**Carotid web**: carotid webs are nonatherosclerotic fibrous bands that arise along the posterior margin of the carotid bulb. They appear on imaging modalities as shelf-like or triangular-shaped intraluminal projections and are thought to be a variant of fibromuscular dysplasia [4]. The prevalence of these lesions is estimated to be on the order of 1% [5]. Nonetheless, they are a relevant entity with regard to stroke, as they are highly thrombogenic.

**CE** (contrast-enhanced): Contrast agents help to distinguish between tissues and to delineate vessels. In CT, they are typically iodine-based, while in MRI, most contrast agents are gadolinium-based.

**CEA** (carotid endarterectomy): Carotid endarterectomy is a surgical procedure performed to remove plaque buildup in the common carotid and internal carotid arteries and improve blood flow.

**ML / DL** (Machine learning / Deep learning): ML describes algorithms that solve tasks without human intervention by being confronted with data. Supervised ML requires labeled datasets, while unsupervised ML identifies patterns within a dataset. DL approaches are a subset of ML. They solve the tasks using so called “artificial neural networks”, that is models consisting of a plethora of “neurons”, which are organized layers. Neurons are processing units that receive, process and pass on information. DL is the foundation for much of the recent progress in medical image processing and can be used for tasks such as detection and segmentation.

**ECST** (European carotid Surgery Trial) The ECST is a multicenter, randomized controlled trial of more than 3000 patients which was performed in the 1980s and 1990s. Its main result was that carotid endarterectomy is indicated for most patients with a recent non-disabling carotid-territory ischemic event, given a carotid stenosis greater than about 80%[6]. The quantification of degree of stenosis in % is defined as “[1- (diameter of most stenotic part / estimated original diameter at site of stenosis)] x 100”. Of note, this definition differs from that used in the NASCET trial.

**FC** (Fibrous cap): The fibrous cap is a layer of fibrous connective tissue. The fibrous cap contains macrophages and smooth muscle cells. When the FC is thin it is prone to rupture and ulceration, which can lead to thrombosis and subsequent ischemic events.

**IPH** (Intraplaque hemorrhage): Intraplaque hemorrhage is a common feature of atherosclerotic plaques and is considered one of the identifying features of complex lesions preceding acute ischemic events. The mechanism by which hemorrhage destabilizes the plaque is largely part secondary to the action of hemoglobin released from red blood cells at the site of the hemorrhage.

**Irregular plaque surface**: Irregularities on the plaque surface with fluctuations between 0.3 mm and 0.9 mm. **It has been demonstrated that these irregularities are a feature indicating plaque vulnerability[7].**

**LRNC** (Lipid-Rich Necrotic Core): LRNC plays a key role in the progression and vulnerability of atherosclerotic plaques. Lipids are the basic components of atherosclerotic plaques. Size of the LRNC in carotid plaques is believed to be a predictive factor for plaque rupture. Previous studies showed the presence of LRNC in carotid atherosclerotic plaque could speed up plaque growth and the progression of luminal stenosis[8].

**NASCET** (North American Stenosis Carotid Endarterectomy trial). Large randomized controlled trial published in 1991 that included more than 2200 patients. It demonstrated that carotid endarterectomy is beneficial to patients with recent nondisabling stroke event. Stenosis quantification was performed using the following formula: “[1 – (narrowest ICA diameter/diameter normal distal cervical ICA)] x 100” [9].

**Near Occlusion**: Carotid near-occlusion describes a distal ICA luminal full or partial collapse beyond a tight stenosis, where the distal lumen should not be used for calculating percentage stenosis. Near-occlusion with full ICA collapse is well-known, with a threadlike lumen [10].

**Remodelling**: Remodelling refers to a change in vessel size (cross-sectional area) in reaction to atherosclerotic changes [11]. In positive or outward remodelling, plaque formation is associated with a compensatory global vessel size increase (total luminal and wall diameter) without luminal diameter change. As plaque growth progresses, eventually the lumen will decrease in size resulting in negative or inward remodelling.

**Stenosis** (degree of): quantification of the stenosis caused by atherosclerotic plaque. It is possible to calculate the stenosis using different approaches: percentage methods (see NASCET and ECST); with lumen area or with lumen diameter.

**Stroke**: Acute neurologic injury caused by either brain ischemia, e.g. due to thrombosis or embolism (~80%), or brain hemorrhage (~20%). It is one of the most important causes of morbidity and mortality worldwide[12].

**Thrombo-embolism:** embolism arising from a fragmented thrombus from the surface of a plaque. This results in large fragments with occlusion of a medium to large vascular bed. It is typically an acute event, uni-lateral and 20-40 times more frequent that an athero-embolism.

**TIA (transient ischemic attack):** The classic definition of TIA is that of focal neurologic symptoms lasting less than 24 hours, caused by a decrease in blood flow. There is an alternative, tissue-based definition: TIA is a transient episode of neurologic symptoms without acute infarction [13].

**TOF** (time-of-flight): TOF is one of the most important non-contrast bright-blood method for imaging the human vascular system. TOF is based the principle of flow-related enhancement, a time-of-flight flow phenomenon where the stationary tissues in an imaged volume become magnetically saturated by multiple repetitive radiofrequency pulses that drive down their steady-state magnetization levels. Blood flowing into the imaged volume has not experienced these pulses and thus has a high initial magnetization. The signal from inflowing blood thus appears bright compared to background tissue.

**Ulceration**: Endothelial defect of at least 1 mm in width with exposure of plaque’s necrotic core to circulation. Carotid plaque ulceration is believed to be a marker of previous plaque rupture as well as a potential thromboembolic source.

**Vulnerable plaque:** this term is used to indicate a plaque which has certain characteristics which increase rupture risk, independent of the degree of luminal stenosis. Composition components of a vulnerable plaque typically include a lipid rich core, a thin fibrous cap with fissure, and intraplaque hemorrhage. Ulceration with irregular surface contour and the presence of inflammation are also associated with an increased rupture risk.

**References**:

1 Johnston KW, Rutherford RB, Tilson MD, Shah DM, Hollier L, Stanley JC. Suggested standards for reporting on arterial aneurysms. J Vasc Surg. 1991 Mar;13(3):452–8.

2 Garg K, Rockman CB, Lee V, Maldonado TS, Jacobowitz GR, Adelman MA, et al. Presentation and management of carotid artery aneurysms and pseudoaneurysms. J Vasc Surg. 2012 Jun;55(6):1618–22.

3 Patra DP, Welz ME, Krishna C, Abi-Aad KR, McClendon J, Turkmani A, et al. Extracranial Carotid Artery Aneurysms. Carotid Artery Disease. Cham: Springer International Publishing; 2020; pp 173–88.

4 Haussen DC, Grossberg JA, Bouslama M, Pradilla G, Belagaje S, Bianchi N, et al. Carotid Web (Intimal Fibromuscular Dysplasia) Has High Stroke Recurrence Risk and Is Amenable to Stenting. Stroke. 2017;48(11):3134–7.

5 Wojcik K, Milburn J, Vidal G, Steven A. Carotid webs: Radiographic appearance and significance. Ochsner J. 2018 Jun;18(2):115–20.

6 Warlow C, Farrell B, Fraser A, Sandercock P, Slattery J. Randomised trial of endarterectomy for recently symptomatic carotid stenosis: Final results of the MRC European Carotid Surgery Trial (ECST). Lancet. 1998;351(9113):1379–87.

7 Saba L, Anzidei M, Marincola BC, Piga M, Raz E, Bassareo PP, et al. Imaging of the carotid artery vulnerable plaque. Cardiovasc Intervent Radiol. 2014;37(3):572–85.

8 Underhill HR, Yuan C, Yarnykh VL, Chu B, Oikawa M, Dong L, et al. Predictors of surface disruption with MR imaging in asymptomatic carotid artery stenosis. AJNR Am J Neuroradiol. 2010 Mar;31(3):487–93.

9 Fowler JF. Beneficial Effect of Carotid Endarterectomy in Symptomatic Patients with High-Grade Carotid Stenosis. N Engl J Med. 1991 Aug;325(7):445–53.

10 Johansson E, Fox AJ. Carotid near-occlusion: A comprehensive review, part 1 - Definition, terminology, and diagnosis. Am J Neuroradiol. 2016;37(1):2–10.

11 Glagov S, Weisenberg E, Zarins CK, Stankunavicius R, Kolettis GJ. Compensatory Enlargement of Human Atherosclerotic Coronary Arteries. N Engl J Med. 1987 May;316(22):1371–5.

12 Katan M, Luft A. Global Burden of Stroke. Semin Neurol. 2018;38(2):208–11.

13 Easton JD, Saver JL, Albers GW, Alberts MJ, Chaturvedi S, Feldmann E, et al. Definition and evaluation of transient ischemic attack: A scientific statement for healthcare professionals from the American heart association/American stroke association stroke council; council on cardiovascular surgery and anesthesia; council on cardiovascular radiology and intervention; council on cardiovascular nursing; and the interdisciplinary council on peripheral vascular disease. Stroke. 2009 Jun;40(6):2276–93.
